# Parallel Droplet Deposition via a Superhydrophobic Plate with Integrated Heater and Temperature Sensors

**DOI:** 10.3390/mi11040354

**Published:** 2020-03-28

**Authors:** Marcus A. Hintermüller, Christina Offenzeller, Marcel Knoll, Andreas Tröls, Bernhard Jakoby

**Affiliations:** Institute for Microelectronics and Microsensors, Johannes Kepler University Linz, 4040 Linz, Austria; christina.offenzeller@jku.at (C.O.); marcel.knoll@jku.at (M.K.); andreas.troels@jku.at (A.T.); bernhard.jakoby@jku.at (B.J.)

**Keywords:** screen printing, droplet evaporation, dosage, resistance temperature detector, heated substrate

## Abstract

A simple setup, which is suitable for parallel deposition of homogenous liquids with a precise volume (dosage), is presented. First, liquid is dispensed as an array of droplets onto a superhydrophobic dosage plate, featuring a 3 × 3 array of holes. The droplets rest on these holes and evaporate with time until they are small enough to pass through them to be used on the final target, where a precise amount of liquid is required. The system can be fabricated easily and operates in a highly parallel manner. The design of the superhydrophobic dosage plate can be adjusted, in terms of the hole positions and sizes, in order to meet different specifications. This makes the proposed system extremely flexible. The initial dispensed droplet mass is not significant, as the dosing takes place during the evaporation process, where the dosage is determined by the hole diameter. In order to speed up the evaporation process, microheaters are screen printed on the back side of the dosage plate. To characterize the temperature distribution caused by the microheaters, temperature sensors are screen printed on the top side of the dosage plate as well. Experimental data regarding the temperature sensors, the microheaters, and the performance of the setup are presented, and the improvement due to the heating of the dosage plate is assessed. A significant reduction of the total evaporation time due to the microheaters was observed. The improvement caused by the temperature increase was found to follow a power law. At a substrate temperature of 80 °C, the total evaporation time was reduced by about 79%.

## 1. Introduction

Liquid droplets are utilized in many different ways in a wide variety of applications [1]. Their use is especially suitable for biochemical analysis, as only a small amount of volume is required to perform the required task [2,3].

In this work, we present a facile setup, which is capable of parallel droplet deposition with precise droplet volume (dosage). The core functionality is based on evaporation of droplets resting on a superhydrophobic hole plate, the concept of which we recently introduced [4,5]. The system can be fabricated using simple methods and is highly parallelizable, allowing for large-scale droplet deposition. In Tröls et al.’s research [4], the working principle was first introduced where evaporation was not aided by heating of the substrate. Measurements were performed with different diameters of the holes of the dosage plate showing the applicability of the underlying principles. Also, considerations of droplet shape during transition through the hole and the dependence of droplet mass on the hole diameter were presented. In addition to the previously reported setup, here, microheaters are implemented on the bottom surface of the device, in order to accelerate the dosing process. Research concerning the evaporation of sessile droplets on heated substrates revealed that the decrease in total evaporation time due to the heating follows a power law trend [6,7]. Assuming similar conditions are present with our setup, a significant reduction in evaporation time can be expected due to the implementation of the microheaters. Also, resistance temperature detectors (RTD) are deposited on the top surface to monitor the temperature during the entire application process. Preliminary results regarding the use of screen printed microheaters were presented in Hintermüller et al.’s research [5]. The additional implementation of temperature sensors directly on the dosage plate allows for a more reliable measurement of the temperature of the plate. This aids the quantification of the reduction in total evaporation time depending on temperature and allows comparison to similar findings in literature.

The proposed system was introduced for the potential application of coating of microneedle arrays for transdermal drug delivery systems by exact droplet deposition [4]. Here, the surface of the microneedles needs to be coated with a precise amount of the drug. Simply dip-coating the microneedle array may lead to patchy coverage [8], and other methods such as electrohydrodynamic atomization, ink-jet printing, and jet- and spray drying require special and often expensive equipment [9,10,11].

When using microtiter plates, for example, for enzyme-linked immunosorbent arrays (ELISA) [12,13], parallel and precise dosing is also required. Instead of using multichannel pipettes, the proposed system may be utilized for this task by directly depositing the required liquid volume as a droplet into the wells.

So-called dispensed droplet systems require deposition of sessile droplets in a well-ordered manner on a substrate to create a planar droplet array [14,15,16]. Here, the proposed setup may also be advantageous to replace the manual deposition of droplets with a predefined volume.

In the current state, the presented system is comparable to conventional pipettes. When working with, for example, microwell plates, using single channel pipettes can be tedious, as the same operation must be performed hundreds of times. Multichannel pipettes improve that situation but are often less accurate and precise. Also, these conventional systems are prone to human error, as it is not uncommon to lose track during repetitions [17]. Such errors not only cost time, but also the sample material is wasted, which might be scarce. This can be improved by using an automated liquid handling system, as such a system can perform many pipetting steps in a short amount of time, operating with robotic precision. Unfortunately, such systems are usually very expensive. The proposed setup is an alternative to the aforementioned systems. Its fabrication is simple, as well as cost effective, and it works semi-automatic, helping to reduce human error, while the dosing performance is comparable to the methods mentioned above.

With the evolution of microfluidics, the employed mechanisms were also utilized for pipetting and dosing methods. Huang et al. developed a hybrid polydimethylsiloxane (PDMS)-glass chip with a series of pneumatic valves to perform precise dosing processes [18]. A collection reservoir was filled by repeatedly combining one of three predefined volumes that could be set with the device. After achieving the desired dosage in the reservoir, it could be dispensed for further usage. More recently, an improved version of a standard micropipette was reported [19,20]. Standard pipettes become increasingly unreliable at volumes below 1 µL, featuring a precision of about 20% or worse [20]. Researchers modified a standard micropipette and combined it with a microfluidic PDMS chip, featuring a flexible membrane. After filling the pipette cartridge, an electromagnetic actuator pushes on the PDMS membrane to release a defined dosage. Using this approach, precision was improved to about 5% in the range of 100 nL–1000 nL. Additionally, the system is programmable, helping to reduce human error.

Over the past years, the analysis of single cells became an important topic in cell biology. Therefore, there was a demand for single-cell dispensation systems. In Yusof and Gross et al.’s researches [21,22], a system for single-cell dispensation was presented. It consisted of a microfluidic dispenser chip, which was filled with fluid and cells through an inlet. Through piezo actuation (similar to a regular inkjet printer) on a deformable silicon membrane, picoliter-sized droplets were generated. An optical system at the end of the nozzle of the dispenser chip was able to detect if a single cell was contained inside the droplet. If so, the droplet was dispensed into a microtiter plate; if not, the droplet was sucked away using a high-speed pneumatic shutter system, which was also located at the end of the nozzle. As of now, commercially available single-cell dispenser systems are made by, for example, NanoCellect Biomedical, Cellenion or Namocell.

We consider the presented system to be appealing due to its simple fabrication, high adaptability, and parallelization. It can be easily adjusted to meet required specifications for certain tasks regarding droplet positioning (by, e.g., changing distances between the holes in the array or even organizing them in completely different patterns) and dosing (by adjusting the hole diameters; also, different sized holes in one plate are possible). The hole plates can be swapped out readily, in order to adjust to varying specifications, making the setup highly flexible. The system can also be scaled up easily to support many droplets at once.

## 2. Principle of Operation

The terminology introduced in Tröls et al.’s research [4] will be adopted here, which means that a distinction between “dispensation” (i.e., rough amount of liquid volume) and “dosage” (i.e., precise amount of liquid volume) will be made.

**Figure 1:**
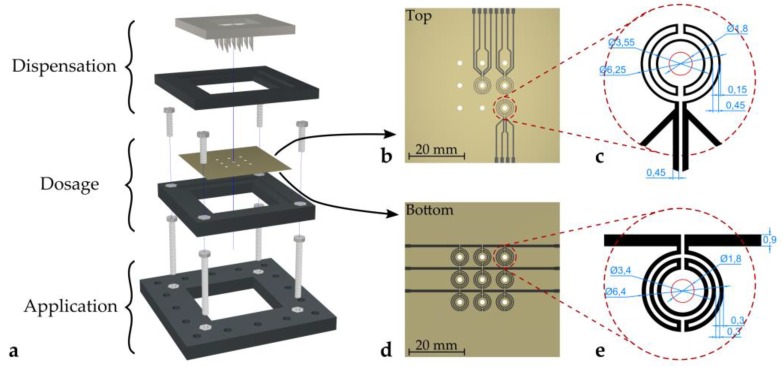
Schematic of the deposition setup. (**a**) Exploded view of the setup with three separate stages (dispensation, dosage, and application). (**b**) Layout of the temperature sensors on the top side of the dosage plate. (**c**) RTD design and dimensions (in mm). (**d**) Layout of the microheater array on the bottom side of the dosage plate. (**e**) Microheater design and dimensions (in mm). Parts (a), (d), and (e): © 2019 IEEE. Reprinted, with permission, from Hintermüller et al. [5].

Figure 1a shows the full setup of the proposed deposition concept. The system can be divided into three separate stages, (i) the dispensation stage, (ii) the dosage stage, and (iii) the application stage. The dispensation stage features a 3D-printed hollow needle array, whereas the dosage stage is comprised of a metal plate featuring an array of holes.

**Figure 2:**
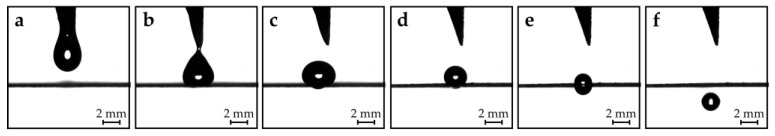
Operational principle. (**a**) and (**b**): Dispensing of one droplet onto the superhydrophobic hole plate. (**c**) and (**d**): Evaporation of the droplet. (**e**) and (**f**): A sufficiently small droplet passes through the hole. Figure 3 from [4] by Tröls et al., used under CC BY/Individual parts rearranged.

The principle of operation is illustrated in Figure 2. The topside of the hollow needle array structure features a reservoir with individual small wells for each needle. If liquid is placed in the reservoir (e.g., by pipetting or simply pouring), liquid is sucked into the hollow needle channels by capillary forces. If enough liquid is placed in the reservoir, gravitational forces can overcome the capillary forces and a single droplet slides along the needle tip. Eventually, the droplet detaches from the tip and subsequently lands on a hole of the metal plate (dosage pate) beneath (Figure 2a,b). The remaining liquid remains inside the capillaries, as long as no additional liquid is placed in the reservoir. During this step, only an approximate amount of the required dosage needs to be dispensed from the needles. The channels of the hollow needles in this study have a diameter of 1.8 mm. The dispensed droplets have an initial mass of about 15 mg.

The precise dosing takes place in the dosage stage as follows. Sessile droplets resting on a plane behave differently during evaporation, depending on the properties of the surface. In general, evaporation takes place in three stages. First, (i) in a constant contact radius (CCR) mode (contact radius remains constant and contact angle decreases), followed by (ii) a constant contact angle (CCA) mode (contact angle remains constant and contact radius decreases), and lastly, (iii) in a mixed mode (both contact radius and contact angle decrease) [23,24,25,26]. For our purposes, the CCA mode is the preferred one, because the droplets need to reduce in diameter to eventually be able to pass through the hole. However, at the beginning of the evaporation process, the droplets evaporate in a CCR mode due to contact line pinning resulting from contact angle hysteresis [27,28,29]. In order to minimize the unwanted evaporation in CCR mode, a superhydrophobic surface with low contact angle hysteresis is required [30]. Usually, this is achieved by structuring the surface, so the droplets rest in a suspended Cassie–Baxter state [31]; the contact angle hysteresis of such surfaces is insignificant [32]. Droplets resting on a superhydrophobic hole were observed to evaporate in a mixed mode [33], but the superhydrophobicity is still required to avoid contact line pinning. The droplets dispensed from the needle array rest on the superhydrophobic hole plate and shrink in size due to evaporation (Figure 2c,d). If the sizes of the droplets eventually match the size of the holes, the droplets are pulled through the hole by gravitational forces (Figure 2e,f). The precisely dosed droplets can then be used within the application stage. High-speed camera recordings of the dispensing and dosing process can be found in the supplementary materials provided in Tröls et al.’s research [4].

For a given liquid, the mass of the dosed droplets is defined only by the geometry of the holes, resulting in the desired exact dosage of the liquid. Different liquids may not necessarily be dosed with the same mass, as fluid properties can affect the dosing process. Liquids with higher density have a higher mass at the same size as lower density liquids. In such a case, gravitational forces are higher; therefore, it can be expected that the liquid droplet is pulled earlier through the hole than it would be for lower density liquids. As discussed in Tröls et al.’s research [4], the droplet’s shape is not perfectly spherical when it passes through the hole. For bigger hole sizes, the deviation from a sphere was higher, which was caused by the increased gravitational forces due to bigger droplets. Similar reasoning can be applied to higher density liquids. As the system relies on evaporation in the CCA mode, contact line pinning (caused by contact angle hysteresis) needs to be avoided as this would inhibit the principle of operation. Being able to evaporate in the CCA mode usually requires high initial contact angles. Therefore, also the surface tension of the liquid can influence the performance of the setup. The evaporation is mainly diffusion driven. Therefore, the diffusion coefficient of the vapor in the gas phase greatly effects the total evaporation time [34]. 

In order to speed up this evaporation process, microheaters were applied by screen printing on the bottom side of the hole plate (schematics are depicted in Figure 1d,e). As heat is transferred from the plate to the droplets, the liquid is warmed up, leading to an increased evaporation flux [35]. Additionally, temperature sensors were applied to the top surface of the hole plate (schematics are depicted in Figure 1b,c) to monitor the temperature of the dosage plate. The microheaters were designed to be radially symmetric to provide uniform heating of a droplet. The linewidth was chosen to ensure reasonable resistance to be able to apply power using a voltage source. The RTD design was chosen accordingly to provide the same symmetry as the microheaters. Linewidths were designed to be narrower in order to achieve a higher resistance, which simplifies the measurement. Compared to, e.g., using a Pt100 temperature probe, which gives only pointwise information, the distributed design of the presented temperature sensor yields a mean temperature around the hole. This value allows for better comparison, as this is also the value a droplet experiences.

With the presented evaporation-based system, exact dosing can be ensured for homogenous liquids. If solutions evaporate, the solute concentration increases as the solvent evaporates. In some applications, the increased concentration may be unacceptable. Nevertheless, it was demonstrated in Hernandez-Perez et al.’s research [14], that for dispensed droplet systems, a preconcentration step caused by solvent evaporation, can be beneficial, especially for samples with low abundance.

## 3. Fabrication

The frames of the three stages of the setup shown in black in Figure 1a were fabricated using a standard desktop 3D-printer (Ultimaker 3 extended, Ultimaker, Utrecht, The Netherlands). The hollow needle array was also 3D-printed (Stratasys Objet 30 Pro, Stratasys Ltd., Eden Prairie, MN, USA).

**Figure 3:**
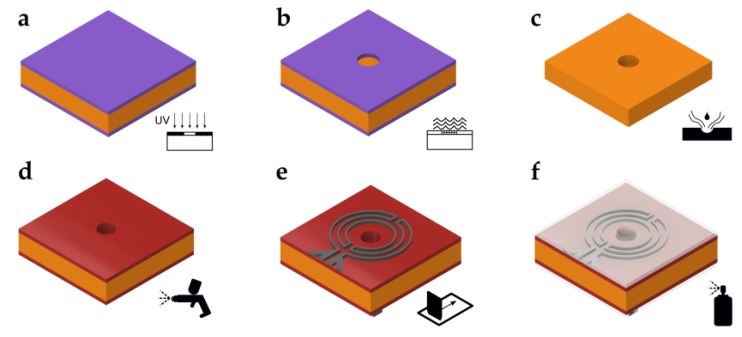
Fabrication process of the dosage plate. (**a**) UV-light exposure. (**b**) Development of photoresist. (**c**) Wet etching. (**d**) Insulation of metal plate. (**e**) Screen printing of heaters and sensors. (**f**) Application of superhydrophobic coating.

**Figure 4:**
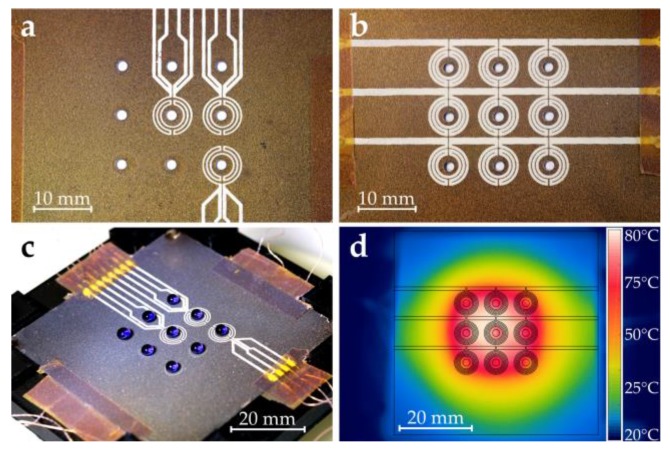
Fabricated dosage plate. (**a**) Close-up of RTDs. (**b**) Close-up of microheaters. (**c**) Fully loaded superhydrophobic dosage plate (droplets are DI water colored with blue ink). (**d**) Infrared camera picture of temperature distribution at 4 V. Note that the outline of the heater layout was overlaid to indicate the heater position. Part (d): ©2019 IEEE. Reprinted, with permission, from Hintermüller et al. [5].

Figure 3 shows the fabrication process of the superhydrophobic hole plate. Starting with a 100 µm thick nickel silver (or German nickel) plate (size 55 mm × 55 mm), which was coated with photoresist on both sides, the hole layout was transferred by a standard photolithography process (Figure 3a). After development of the photoresist (Figure 3b), the transferred pattern was etched away, leaving the holes in the metal plate (Figure 3c). In this example, a 3 × 3 array of holes with a diameter of 1.8 mm was etched. Next, the metal plate was insulated to avoid shorting of the microheaters and RTDs (Figure 3d). To this end, an insulation coating, made from 33 wt% Rhodeftal 210 ES (Huntsman International LLC, The Woodlands, TX, USA), 40 wt% N-methyl-2-pyrrolidone (NMP), and 27 wt% p-xylene, was prepared. After mixing of the components, 0.1 wt% of the surface additive BYK 310 (BYK Additives and Instruments, Wesel, Germany) was also incorporated to reduce the surface tension of the coating [36,37]. Using a spray coating process, the insulating coating was applied to the top and bottom surfaces. Between layer applications, the plate was preheated on a hotplate to approximately 230 °C and finally cured at 250 °C for approximately 10–15 min. Next, the microheaters and RTDs were applied by the screen printing process (Figure 3e). First, the RTDs were printed using a 180 threads/cm PET (polyethylene terephthalate) mesh and then the microheaters, using a 100 treads/cm PET mesh. A conductive polyimide silver ink (KA 801, DuPont, Wilmington, DE, USA) was used in both cases. After each printing step, the ink was cured for about 15 min on a hotplate at about 150 °C. Lastly, the top and bottom sides of the dosage plate were coated using the commercially available impregnation spray NeverWet (Rust-Oleum, Vernon Hills, IL, USA) following the guidelines provided by the manufacturer (Figure 3f). Using this spray, the contact angle between a plane and water was measured to be about *θ*_0_ = 154° after coating [38]. Figure 4a,b show close-up photographs of the fabricated RTDs and microheaters on the dosage plate after applying the superhydrophobic coating, respectively.

Figure 4c shows a picture of the fully loaded dosage plate. Note that deionized (DI) water mixed with blue ink was used for this picture to enhance contrast. The measurements below were performed using only DI water.

Each of the microheater meanders had an electrical resistance of about 4.5 Ω. The three traces of the microheater design were connected in parallel via the connection cables. Figure 4d shows a thermal image (FLIR E4, FLIR Systems Inc., Wilsonville, OR, USA) of the topside of the dosage plate after applying DC voltage (4 V) to the heaters. Since the heat distribution is not uniform, droplets in the center of the plate tend to evaporate faster than corner droplets due to the higher temperature in the center. The layout of the RTDs was designed in a way to capture all tree temperature zones, ranging from the hottest area (center) to slightly cooler parts (in the middle of the sides) to the sections with the lowest temperature (corners). Considering the symmetry of the temperature distribution, these three points were sufficient to characterize the temperature conditions of the dosage plate.

## 4. Results

### 4.1. Sensor Characterization

**Figure 5:**
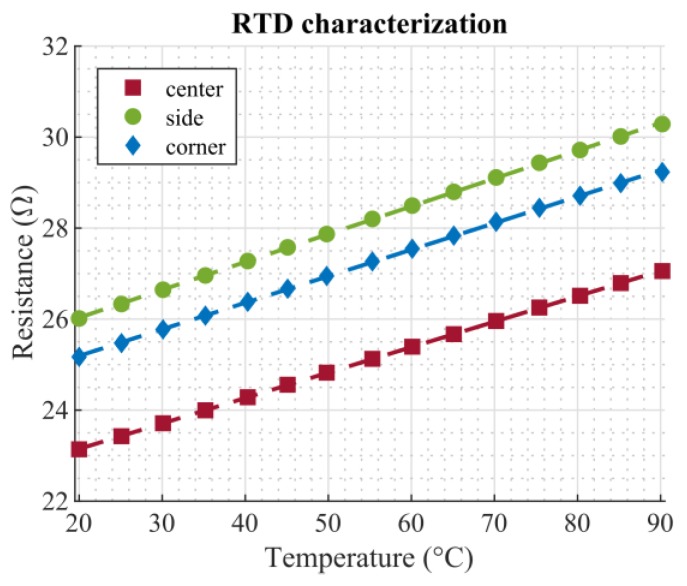
Calibration curve of the RTDs. The markers show measurement points, the dashed lines corresponding linear fits.

RTD characterization was done by measurements in a climate chamber (Weiss WKL 100, Weiss Technik, Reiskirchen, Germany). To this end, the setup was put inside the climate chamber, and a temperature sweep ranging from 20 °C to 90 °C with a step size of 5 °C was performed. Each temperature step was held constant for 35 min, allowing the chamber to reach a steady state. The relative humidity was kept at a constant level of 50%. The resistance of the three RTDs was measured using a 4-wire configuration in order to eliminate any influence of the connection wires and traces. Measurements were performed using digital multimeters (Keithley DMM7510, Keithley Instruments LLC, Solon, OH, USA). Figure 5 shows the measured calibration curves. In the considered temperature range, the characteristics of the RTDs can be described by a linear relationship. The slopes of the three curves were similar; the offsets of the characteristics can be attributed to inconsistencies in the manual screen printing process and do not affect further measurements. Table 1 lists the fitting parameters for the linear relationship *R* = *k*_1_*T* + *k*_2_, as well as the corresponding coefficients of determination, *r*^2^.

**Figure 6:**
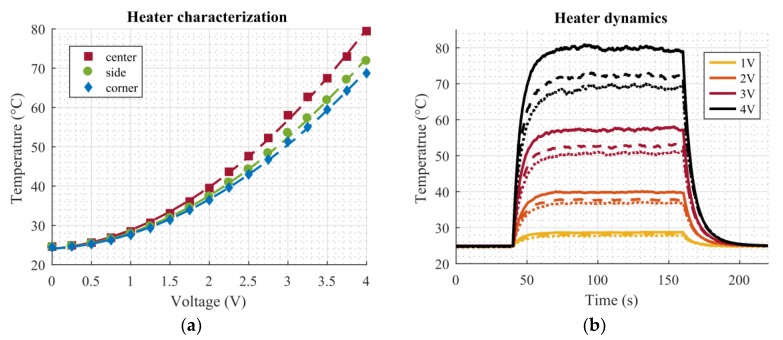
Microheater characterization. (**a**) Microheater characteristics. The markers show measurement points, the dashed lines corresponding quadratic fits. (**b**) Microheater dynamics for several voltage steps. Solid lines correspond to the center position, dashed lines to the side position, and dotted lines to the corner position. The voltage step for all measurements starts at 40 s and ends at 160 s.

The microheaters were characterized by means of a sweep of the applied DC voltage ranging from 0 V to 4 V in 0.25 V steps. These measurements were also conducted inside the climate chamber at a temperature of 25 °C and relative humidity of 50%. Again, the resistance of the temperature sensors was measured using the same setup as described above. Figure 6a shows the measured characteristics of the microheaters. The measured resistance of the temperature sensors was already converted to the corresponding temperature using the calibration curves from Figure 5. Since the microheaters work by Joule heating, a quadratic dependency on the applied voltage can be expected, as can also be seen in Figure 6a. Corresponding fitting parameters of the quadratic curves (*T* = *p*_1_∙*V*^2^+ *p*_2_∙*V* + *p*_3_) are listed in Table 2. As mentioned before, it is evident from Figure 4d that the highest temperature on the dosage plate occurs in the center, the sides are slightly cooler, and the corners are the coldest areas. From Figure 6a, it can be seen that this difference becomes more pronounced at higher temperature increases, as the curves deviate significantly for applied voltages above 2 V in Figure 6a.

Next, the dynamics of the heaters and the achievable steady state temperature at several applied voltages were investigated. Again, the setup was put inside the climate chamber at *T* = 25 °C and *RH* = 50%. Voltage steps of 1 V, 2 V, 3 V, and 4 V were applied for 120 s in order to approach the steady state. Figure 6b shows the measurement results. The voltage step was switched on at the 40 s mark and switched off at 160 s. Again, the temperature was calculated from the RTD resistances. The temperature differences between the center (solid lines), side (dashed lines), and corner (dotted lines) can also be clearly seen in this plot. At an applied voltage of 4 V, the difference between the center and corner positions is about 10 °C. From Figure 6b, the maximum temperatures caused by the four different voltage levels were determined as 29 °C at 1 V, 40 °C at 2 V, 57 °C at 3 V, and 80 °C at 4 V. 

**Figure 7:**
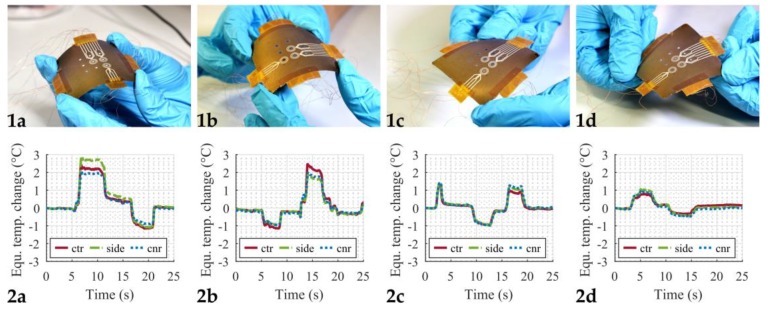
Bending tests. (**1a**–**1d**) Pictures of the manually applied deformation of the dosage plate. (**2a**–**2d**) Equivalent temperature change caused by bending, calculated from the resistance change of the RTDs. Legend: Solid red lines correspond to the center position (ctr), dash-dotted green lines to the side position (side), and dotted blue lines to the corner position (cnr).

As the dosage plate is heated by the microheaters on the back side, the plate can bend slightly due to thermal expansion of the material. Since the RTDs also change their resistance due to deformation (then acting as strain gauges), several bending tests were performed in order to rule out the spurious impact of this deformation on the measured temperature. To do so, the dosage plate was manually bent, and the resistance change was measured. Figure 7 shows the results from these bending tests. The top row (Figure 7(1a–1d)) shows the four different bending configurations that were investigated. For all four measurements, at least one upward and one downward bending motion was performed. The change in resistance of the RTDs was converted to an equivalent temperature change using the calibration curves from Figure 5. Hence, the results in the bottom row (Figure 7(2a–2d)) show an apparent temperature change caused by the bending of the plate and not by an actual temperature increase or decrease. The maximum equivalent temperature change from this experiment was about 3 °C. Considering that this manually forced deformation here was at least ten times higher than what was observed from the thermal expansion, the influence of the plate bending due to the increased temperature is considered to be negligible for our purposes.

### 4.2. Device Performance

Next, the performance of the entire setup was investigated. To this end, mass measurements of released droplets were performed by tracking the mass using a high-precision scale (HR-250 AZ, A&D Company, Tokyo, Japan). The setup was put above the scale, and the whole array of nine droplets (DI water was used in the experiments) was dispended onto the dosage plate. Then, the droplets were left to evaporate. Each time a droplet passed through a hole of the dosage plate and subsequently impinged on the scale, the measured mass of the scale increased. From these measurements the individual mass of each of the droplets was extracted. Measurements were performed for different temperatures to investigate the improvement in evaporation time due to the microheaters. 

**Figure 8:**
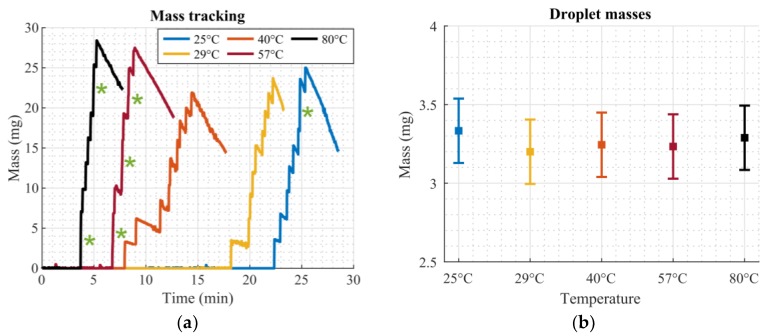
Droplet mass measurements at different heater temperatures. (**a**) Tracked mass for different temperatures. Jumps in mass correspond to droplets impinging on the scale. Green asterisks (*) indicate two droplets impinging on the scale almost simultaneously. (**b**) Error bar plot of droplet masses at different temperatures. Squares show the mean value, error bars show the standard deviation from Table 3.

Figure 8a shows the tracked mass for the different temperature levels. Measurements were performed at a room temperature of 25 °C. The sudden increases in mass were caused by droplets impinging on the scale. Since the initial volumes of the droplets were different, due to the non-exact dispensation step, all nine droplets were not released at the same time, as slightly larger droplets require a longer time to evaporate until the required size was reached. Green asterisks (*) in Figure 8a indicate that two droplets almost simultaneously impinged on the scale; therefore, the measured mass was registered as double the mass of one droplet. The downward slope between the mass jumps was caused by evaporation of the already released droplets on the scale. The measurements were stopped after the ninth droplet was released, as further measurements would not yield significant additional information. 

The mass of each droplet was extracted from the tracked mass measurements given in Figure 8a by calculating the difference in mass before and after a droplet impinged on the scale. In cases where two droplets hit the scale almost simultaneously (green asterisks (*) in Figure 8a), the mean mass of the mass jump was used for both droplets. Table 3 lists calculated masses for each droplet for every measurement from Figure 8a. Also, mean values and standard deviations are given. Figure 8b summarizes these values in an error bar plot. No obvious influence of the increased temperature on the released droplet masses could be identified, demonstrating that the system was capable of dosing an equal amount of liquid with every droplet that passed through the dosage plate. A detailed statistical analysis is provided in Appendix A.

**Figure 9:**
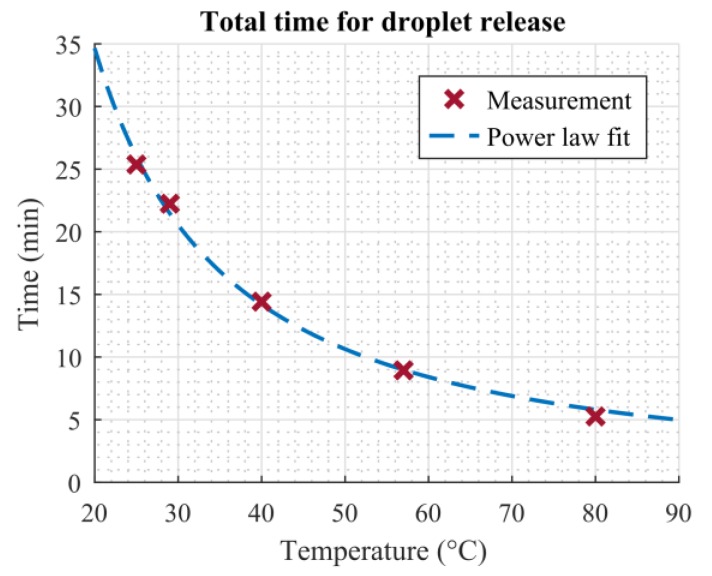
Time required until the last droplet is released vs. temperature. Red crosses show measured data, the dashed blue line shows a power law fit (*t* = *aT^b^*, *r*^2^ = 0.9954).

Figure 9 shows the total time required for the ninth droplet to pass through the dosage plate (red crosses). The times plotted were taken from Figure 8a and correspond to the moment the last droplet of each measurement impinged on the scale. A significant decrease in evaporation time, until the last droplet was released, can be seen. Even the smallest temperature increase is already visible in the total evaporation time. Girad et al. investigated the evaporation of sessile droplets resting on a heated substrate using numerical simulations. They found that the relationship between the total evaporation time of a droplet and temperature could be described by a power law [6]. Later, Dash and Garimella were able to confirm these findings in their experiments [7]. As our setup shares similarities with the evaporation of sessile droplets evaporating on heated surfaces, it is assumed that the relationship between total evaporation time and heater temperature can also be described using a power law relation. The blue dashed line in Figure 9 shows a fit of the measurement points using the relation *t* = *a*∙*T^b^*. The fitting parameters were calculated as *a* = 1647 and *b* = 1.289. The power law fit is in good agreement with the measured data.

## 5. Discussion and Conclusions

This work presented a simple setup which is suitable for precise deposition and dosing of homogenous liquids. The dosing was performed by means of droplet evaporation on a superhydrophobic hole plate. Additionally, screen-printed microheaters on the bottom side of this plate were utilized to accelerate the evaporation process significantly without compromising the functionality of the system. The improvement of the total evaporation time could be described by a power law, which is in agreement with findings in the literature concerning similar problems. The relative improvement in total evaporation time due to the heated plate, compared to evaporation at room temperature, was 12.34% (at 29 °C), 43.16% (at 40 °C), 64.76% (at 57 °C), and 79.27% (at 80 °C). The addition of screen-printed temperature sensors helped to monitor the temperature directly on the superhydrophobic plate. 

The precision of the presented system can be compared with commercially available systems by introducing the coefficient of variation *c*_v_ (which is defined as the ratio of the standard deviation to the mean value). From Table 3, this value was calculated being about 6% for the proposed setup. The performance is compared to commercially available single channel and multichannel pipettes (brand name: Eppendorf Research® plus, Eppendorf, Hamburg, Germany) and an automated liquid handling system (brand name: epMotion® Dispensing Tools, Eppendorf, Hamburg, Germany). Values were taken from operating manuals of the manual pipettes [39] and the automated system [40], respectively. For single channel pipettes with variable volume between 0.5 µL–10 µL at a test volume of 5 µL, the *c*_v_ value was found to be 0.8%, and for pipettes with variable volume between 2 µL–20 µL at a test volume of 2 µL, the value is 1.5%. Multichannel pipettes with variable volume between 0.5 µL–10 µL with 8 or 12 channels have a *c*_v_ value of 2% at a test volume of 5 µL, and ones with 16 or 24 channels and volumes between 1 µL–20 µL at a test volume of 2 µL have a *c*_v_ value of 5%. Lastly, the single tip tool (1 µL–50 µL) and the multi tip tool (0.2 µL–10 µL, 8 channels) of the automated system have *c*_v_ values of 3% and 2%, respectively, both at test volumes of 5 µL. The performance of the presented setup is comparable with already commercially available systems.

The dosed mass can be adjusted by changing the hole diameter for a given liquid. In Tröls et al.’s research [4], the dosed mass was measured for varying hole diameters. No obvious change in precision due to the differently sized holes was observed. For small hole diameters, and therefore corresponding small droplets, the dosed mass tends to approach the expected mass of a perfectly spherical droplet. Gravitational forces are so low that the droplet is required to evaporate until the hole diameter is met, at which time it can pass through the hole. For larger hole diameters, gravitational forces help pull the droplets through the hole by slightly squeezing them. Such droplets take on an oval shape during transition through the hole. Their mass is higher than that of a spherical droplet of the same diameter as the hole. The accuracy of the present system is mainly affected by the fabrication process. Misalignments of the photomasks on the top and bottom of the metal plate during UV exposure can result in holes that deviate from a perfectly shaped circle after the etching process. Also, uneven etching can have an effect on the hole geometry. Lastly, uneven application of the insulation and especially the superhydrophobic coating can affect accuracy and precision negatively. Most of these shortcomings could be potentially improved by an automated fabrication process, as in this work each step was performed manually. 

The temperature measurements confirmed that the temperature distribution is not uniform across the hole plate. A hotspot was prominent in the center of the plate. This might become an issue when liquids are to be dosed, that should not be heated above a certain temperature. Although the supplied power could be lowered, in order to reduce the temperature of the hotspot to an allowed level, this would impair the overall performance of the device in terms of evaporation time, as the rest of the dosage plate would also be cooler. Adjusting the design, by, for example, reducing the resistance of the center microheater (e.g., by broadening the printed lines), would probably result in a more uniform temperature distribution. Then, the entire plate could be operated at the maximum allowed temperature, resulting in the shortest possible evaporation time. 

The combination of microheaters and temperature sensors, which can be processed directly onto the plate, can be used to further study the evaporation behavior of droplets resting on a superhydrophobic hole on a heated substrate. Results obtained using this approach could be compared to models considering the evaporation of sessile droplets.

The proposed system is captivating due to its simple implementation. It can be easily scaled up and is therefore capable of highly parallelized droplet deposition and dosing processes. Additionally, the setup is flexible, as the position and size of the holes of the superhydrophobic dosage plate can be adjusted straightforwardly in order to meet required specifications. By simplify swapping out the dosage plate, the setup can be adjusted for a different task very quickly. The proposed system can therefore be of particular interest for various automated or parallelized tasks in biochemical laboratories, e.g., the coating of microneedle arrays, liquid handling on microtiter plates or for dispensed droplet systems.

## Figures and Tables

**Table 1:** Fitting parameters of calibration curves.

Position	*k*_1_ (Ω/°C)	*k*_2_ (Ω)	*r* ^2^
Center	0.0559	22.03	0.99996
Side	0.0611	24.81	0.99986
Corner	0.0583	24.03	0.99975

**Table 2:** Fitting parameters of heater characteristics.

Position	*p*_1_ (°C/V^2^)	*p*_2_ (°C/V)	*p*_3_ (°C)	*r* ^2^
Center	3.061	1.726	24.053	0.99925
Side	2.686	1.382	24.069	0.99936
Corner	2.523	1.247	24.063	0.99954

**Table 3:** Measured droplet masses.

	Droplet Masses (mg)				
Droplet Number	*T* = 25 °C	*T* = 29 °C	*T* = 40 °C	*T* = 57 °C	*T* = 80 °C
1	3.60	3.50	3.30	3.40	3.55
2	3.50	3.40	3.20	3.40	3.55
3	3.60	3.30	3.60	3.20	2.90
4	3.20	3.00	3.20	3.35	3.30
5	3.40	3.40	3.30	3.35	3.30
6	3.20	3.00	3.40	3.20	3.30
7	3.25	3.10	3.20	3.00	3.20
8	3.25	3.00	3.10	3.20	3.20
9	3.00	3.10	2.90	3.00	3.3
$\bar{m}\pm \sigma_{m}$	3.33 ± 0.20	3.20 ± 0.20	3.24 ± 0.19	3.23 ± 0.16	3.29 ± 0.19

## Appendix A

### Statistical Analysis of Droplet Mass

In order to rule out any potential influence of the increased temperature due to the heaters on the droplet masses, analysis of variance (ANOVA) was performed [41]. Using this tool, the significance of the null hypothesis

(1) $$H_{0}={\bar{m}}_{1}={\bar{m}}_{2}=\cdots ={\bar{m}}_{k}$$

meaning that there is no difference between the mean values of the masses of the five measurements at different temperatures, could be estimated.

First, the explained sum of squares (due to a potential temperature influence)

(2) $$SS_{E}=\overset{k}{\underset{i=1}{\sum }}n_{i}{\left({\bar{m}}_{i}-\bar{M}\right)}^{2}=0.097$$

and the sum of squares of residuals (e.g., errors)

(3) $$SS_{R}=\overset{k}{\underset{i=1}{\sum }}\left(n_{i}-1\right)\sigma_{i}^{2}=1.456$$

were calculated. Here, *k* is the number of groups (*k* = 5, measurements at five temperatures), *n_i_* the number of values in each group (*n_i_* = 9, number of droplets in each measurement), and $\bar{M}$ the mean of mass of all measured values.

Next, the *F*-value was calculated using the mean sum of squares

(4) $$F=\frac{MS_{E}}{MS_{R}}=\frac{SS_{E}/\left(k-1\right)}{SS_{R}/\left(N-k\right)}=0.665$$

where *N* = 45 is the total number of measured values. This value was compared to the critical value of the *F*-distribution evaluated using the degrees of freedom *F*(*k* − 1,*N* − *k*) = *F*(4,40). At a significance level of 5%, the critical value is given as *F*_crit_ = 2.61. Since *F* < *F*_crit,_ the null hypothesis could be accepted, and differences in the mean values were not attributed the increased temperature.

Lastly, the homogeneity of variances was tested, as this is a crucial requirement for the ANOVA. To do so, a post-hoc Levene’s test was performed. Here, the test statistic *L* was calculated as

(5) $$L=\frac{\sum_{i=1}^{k}n_{i}{\left({\bar{Z}}_{i}-\bar{Z}\right)}^{2}/\left(k-1\right)}{\sum_{i=1}^{k}\sum_{j=1}^{n_{i}}{\left(Z_{ij}-{\bar{Z}}_{i}\right)}^{2}/\left(N-k\right)\;}=0.488$$

where $Z_{ij}=\left|m_{ij}-{\bar{m}}_{i}\right|$, ${\bar{Z}}_{i}$ being the mean of $Z_{ij}$ of group *i* and $\bar{Z}$ the mean of all $Z_{ij}$. Considering the same *F*-distribution as above, the *L*-value was below the critical one, revealing homogeneity of variances and applicability of the ANOVA.
